# Barriers and Facilitators to Timely Detection and Optimal Management of Chronic Wet Cough in Aboriginal Children Across Australia From the Perspectives of Carers of Aboriginal Children: A Qualitative Study

**DOI:** 10.5694/mja2.70313

**Published:** 2026-10-01

**Authors:** Gloria T. Y. Lau, Pam Laird, Robyn Aitken, Melanie Barwick, Liam Bedford, Emily R. Bowden, Jamie Everingham, Angela Fuery, Reece Griffin, Neha Jain, Mina Kinghorn, Renae Knapp, Gabrielle B. McCallum, Peter Morris, Richard Norman, Peter C. Richmond, Aarti Saiganesh, Slade Sibosado, Cameron Taylor, Maree Toombs, Roz Walker, Joan Wilson, Anne B. Chang, André Schultz

**Affiliations:** ^1^ The Kids Research Institute Perth Western Australia Australia; ^2^ Perth Children's Hospital Perth Western Australia Australia; ^3^ University of Western Australia Perth Western Australia Australia; ^4^ Flinders University Darwin Northern Territory Australia; ^5^ Hospital for Sick Children Toronto Ontario Canada; ^6^ University of Toronto Toronto Ontario Canada; ^7^ The Kids Kimberley The Kids Research Institute Broome Western Australia Australia; ^8^ Charles Darwin University Darwin Northern Territory Australia; ^9^ Carbal Medical Services Toowoomba City Queensland Australia; ^10^ University of Queensland Brisbane Queensland Australia; ^11^ Royal Darwin Hospital Darwin Northern Territory Australia; ^12^ Curtin University Perth Western Australia Australia; ^13^ Kimberley Aboriginal Medical Service Broome Western Australia Australia; ^14^ Derbarl Yerrigan Health Services Perth Western Australia Australia; ^15^ University of Sydney Sydney New South Wales Australia; ^16^ Murdoch University Perth Western Australia Australia; ^17^ Yura Yungi Medical Service Aboriginal Corporation Halls Creek Western Australia Australia; ^18^ Australian Centre for Health Services Innovation and School of Clinical Medicine Queensland University of Technology Brisbane Queensland Australia

**Keywords:** child health, childhood diseases, primary care, respiratory tract infections

## Abstract

**Objective:**

To identify barriers and facilitators to the timely detection and optimal management of chronic wet cough (CWC) in children from the perspectives of carers, across multiple, diverse First Nations Australian communities.

**Study Type:**

Qualitative participatory action research study.

**Setting and Participants:**

Eight Australian communities across three states and territories. Semi‐structured interviews and focus groups with 167 parents and carers of Aboriginal children were conducted between May 2021 and July 2023.

**Main Outcome Measures:**

Barriers to and facilitators of detection and management of CWC in Aboriginal children in a primary care setting.

**Results:**

Key barriers identified included inadequate health promotion, the common perception that CWC is normal and high turnover among health staff, leading to mistrust of newly onboarded health staff. The overarching facilitator identified was knowledge dissemination through health promotion, delivered in person by Aboriginal health staff using culturally secure resources, supplemented by posters placed in the community and the use of radio and social media. Individual communities expressed varied preferences regarding specific health promotion methods and designs. Communities valued staff who had spent considerable time within the community, allowing for the development of trust and rapport.

**Conclusion:**

The barriers and facilitators to detecting and managing CWC in children were generally consistent across the settings we studied. Implementation approaches to facilitate care require tailoring to local settings. Further, to reduce identified barriers for the detection and management of CWC in children, the incorrect perception that CWC is normal must be addressed and the potential harms of staff turnover need to be recognised.

## Plain Language Summary

**The Known:** Chronic wet cough (CWC), a symptom of several chronic respiratory diseases, is prevalent among First Nations children and can lead to bronchiectasis if untreated.

**The New:** Families from eight communities across three states and territories identified limited health promotion as a major barrier to health seeking for CWC. Families wanted knowledge dissemination through culturally secure health promotion tailored to each community. Communities valued and were more likely to trust regular (as opposed to transient) health staff.

**The Implications:** Tailored approaches that reflect each community's unique characteristics are essential. Reducing staff turnover is vital to improve CWC outcomes.

## 1 Introduction

Prevention of chronic illness, vital for future health outcomes, requires an understanding of the associated barriers and facilitators when treating the precursors of chronic illness. Chronic lung diseases, including bronchiectasis, are prevalent among First Nation Australians [1, 2], and one of its known precursors is protracted bacterial bronchitis (PBB) [3]. PBB occurs in 10% of preschool children in the West Kimberley, Western Australia [4].

Children with PBB have chronic wet cough (CWC); that is, daily wet cough lasting over 4 weeks. The timely detection of CWC enables diagnosis and treatment of PBB and the prevention of bronchiectasis [3]. Our previous work in a regional town in Western Australia found that implementing a targeted program to address identified barriers in detecting and managing CWC improved health seeking, clinician assessment of paediatric respiratory illness and cough‐specific quality‐of‐life [5]. The program consisted of the following seven core components: First Nations lead, stakeholder engagement, local champion, information campaign for families, clinician training, educational resources and health system changes [5].

However, although First Nations communities may share common experiences and values that can inform broader approaches, they can vary widely in culture and geography, each with unique customs and ways of understanding health and wellbeing. Thus, our study aimed to identify barriers and facilitators to detecting and managing CWC in primary care settings from the perspectives of carers of First Nations children in multiple diverse communities.

## 2 Methods

### 2.1 Study Design

We conducted a multi‐centre qualitative study with a participatory action research approach (i.e., an iterative, reflexive methodology that involves participants as collaborators) [6]. Although First Nations Australians consists of both Aboriginal and Torres Strait Islander people, some communities, such as the communities included in this study, are Aboriginal and, therefore, prefer to be called Aboriginal. Hence, we used this term throughout this manuscript when referring to the study population.

Before the research commenced, extensive community engagement was undertaken to ensure the study aligned with local needs and priorities. The research involved semi‐structured interviews and focus groups with parents and carers (hereafter carers) of Aboriginal children, followed by a thematic analysis. Concurrently, we conducted interviews with healthcare providers working with Aboriginal children to capture a diverse range of perspectives; these data were analysed independently and reported in a separate manuscript [7].

The CONSIDER reporting criteria checklist for health research involving Indigenous peoples was completed for this article and can be found in Section S1 [8].

### 2.2 Study Setting

Eight Australian Aboriginal communities participated across three states and territories (Table 1). The communities represented a wide diversity in geography, climate [9, 10, 11] and proportion and size of Aboriginal population, ranging from 2% in a major city of 2 million people to 95% in communities as small as a 100 people [12, 13, 14].

**TABLE 1:** Geographical settings of the study sites.

Community	Region, state	Environment/climate [9, 10, 11]	Total population (aboriginal population) [12, 13, 14]	Number of aboriginal medical service clinics
1	Kimberley, Western Australia	Desert/arid	472 (430; 91%)	1
2^a^	Kimberley, Western Australia	Desert/arid	(111)	1
3	Kimberley, Western Australia	Desert/arid	202 (178; 88%)	1
4	Kimberley, Western Australia	Semi‐arid	3603 (2806; 78%)	1
5	Perth, Western Australia	Mediterranean	2,116,647 (42,083; 2%)	4
6	Victoria Daly, Northern Territory	Tropical	411 (390; 95%)	1
7	East Arnhem, Northern Territory	Tropical	705 (645; 91%)	1
8	Darling Downs, Queensland	Humid subtropical	280,000 (17,000; 6%) [13]	1

^a^ No data were available for the total population in Community 2.

### 2.3 Interview Guide Development

The team's previous interview guides on lung health were used to identify barriers to timely detection and optimal management of CWC (Box S1) [15, 16]. Interviewers adapted the language of the interview guides to tailor use in each community. Interviews also considered barriers and facilitators to program implementation, guided by the Consolidated Framework of Implementation Research (CFIR) [17], and the analysis and results of these findings will be reported elsewhere.

### 2.4 Selection Criteria and Recruitment

The CFIR [17] guided identification of interviewee groups (Table 2). Participants were recruited with the help of Aboriginal community navigators through purposive sampling [18] and snowball techniques [18] with verbal, email or letter invitations to ensure that a wide range of demographics and roles were captured. Recruitment also occurred through posters in the community promoting forthcoming focus groups. The number of participants was estimated using information power [19] and anticipated to be ~5–20 individual interviews per site. Participants were recruited until data saturation [18] was reached for each site.

**TABLE 2:** Stakeholder groups based on relevant Consolidated Framework of Implementation Research (CFIR) domains.

Inner setting (i.e., internal factors within a community): Carers of Aboriginal children Providers of primary care healthcare to Aboriginal children (e.g., doctors, nurses, Aboriginal Health Workers, physiotherapists) Primary care managerial staff (e.g., managers, board members)
Outer setting (i.e., external factors beyond the community): Paediatricians who provide a service to Aboriginal children Policymakers and guideline providers

### 2.5 Data Collection

Interviews and focus groups were conducted between May 2021 and July 2023. Table 3 details the interview periods and interviewers for individual sites. The interviews occurred at different times across sites, as determined by each site's readiness, which was influenced by ethics approvals, governance processes, stakeholder engagement and environmental factors limiting travel to the community.

**TABLE 3:** Interviewer demographics and interview periods for each site.

Communities	Region, state	Qualifications and demographics of interviewers	Time period for qualitative data collection
1	Kimberley, Western Australia	Paediatric respiratory doctor (male) Paediatric respiratory doctor (female) Paediatric physiotherapist with qualitative research expertise (female) Research program manager (female) Aboriginal researcher and cultural navigator (male) Three local Aboriginal community members (female)	May 2021–October 2021
2	Kimberley, Western Australia		
3	Kimberley, Western Australia		
4	Kimberley, Western Australia		
5	Perth, Western Australia	Paediatric physiotherapist (female) Local Aboriginal Health Worker (male) Two local Aboriginal nurses (female)	January 2022–December 2022
6	Victoria Daly, Northern Territory	Research nurse (female) Research nurse with qualitative research expertise (female)	November 2022–May 2023
7	East Arnhem, Northern Territory	Research nurse (female) Research nurse with qualitative research expertise (female) Aboriginal research assistant (female)	June 2023–July 2023
8	Darling Downs, Queensland	Paediatric respiratory doctor (male) Aboriginal qualitative researcher (female)	October 2021–November 2022

Interviews were conducted (usually in pairs) by interviewers with qualitative research skills in Aboriginal settings. An Aboriginal interviewer participated in interviews in all sites except one in the Northern Territory. Informed consent was obtained at the time of the interviews. Interviews occurred in the clinic or out in the community. Handwritten notes or audio recordings were taken of interviews; recordings were subsequently typewritten and notes were compared for any inconsistencies between interviewers, and clarification was sought from interviewees when needed. Interviews typically lasted 5–15 min, with some lasting up to 60 min. Durations were flexible and interviewee guided. Field notes were made during the interview or shortly after when time permitted.

### 2.6 Data Analysis

Qualitative data for each site were analysed individually using a reflexive thematic analysis based on Braun and Clarke's six‐phase approach [20]. The analysis involved familiarisation with the data, generation of initial codes (labels assigned to segments of text that capture their meaning), development and refinement of themes and iterative review and definition of themes to ensure they accurately reflected the interviewees' perspectives from each site. Given the proximity of sites 1–3 and the mobility of Aboriginal families across these three communities, qualitative data for these three sites were combined. Initial site‐specific analyses were undertaken to identify preliminary codes and themes, followed by a cross‐site analysis to enable comparison, ensure consistency in coding and language, and refine overarching themes. At least three researchers performed each site's data analysis, with an example illustrated in Figure 1. The analysis followed an inductive and deductive approach and the research team's previous work directed theme and subtheme conceptualisation [15, 16]. Each analysis phase adhered to Lincoln and Guba's criteria of trustworthiness [21]. The data were managed using QSR NVivo Release 1 [22], with all de‐identified data held on password‐protected institutional servers, in accordance with data governance protocols. After completion of the data analysis, the data were presented to the communities and key decision‐makers, allowing them to review and provide feedback.

**FIGURE 1:**
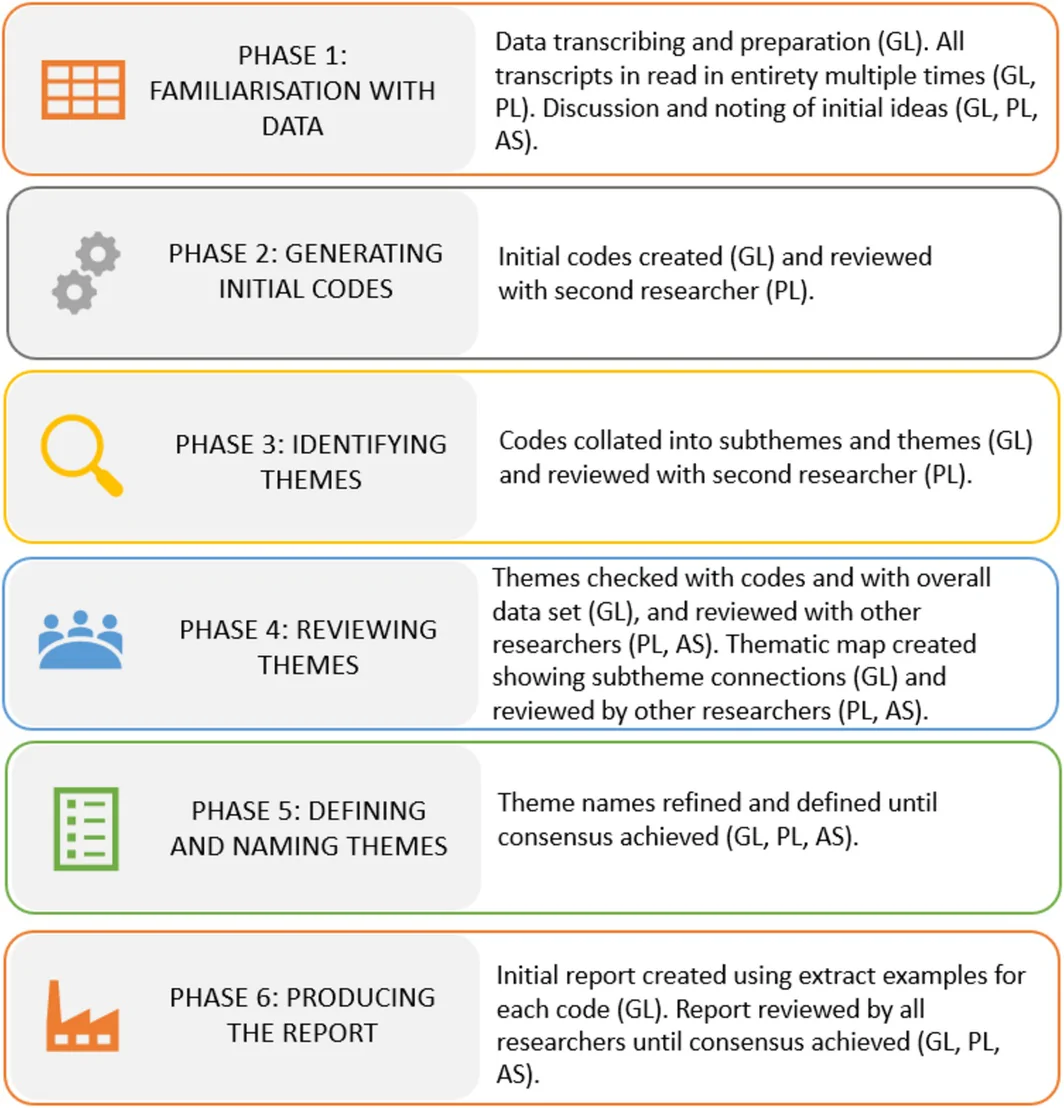
Data analysis process for the East Kimberley sites. AS, André Schultz; GL, Gloria T. Y. Lau; PL, Pamela Laird.

### 2.7 Ethics Statement

Ethics approval for our study was obtained from the Western Australian Child and Adolescent Health Service Ethics Committee (RGS 4136), the Western Australian Aboriginal Health Ethics Committee (HREC 774), the Human Research Ethics Committee of the Northern Territory Department of Health and Menzies School of Health Research (HREC 2021‐3954), the Miwatj Research and Ethics Advisory group, and Reciprocal ethics was given by the University of Queensland Human Research Ethics Committee (2020002563).

We reported our study according to the Consolidated Criteria for Reporting Qualitative Research (COREQ; Table S1) [23].

## 3 Results

The number of participants across the diverse demographics and cultural communities varied (total number of participants, 167; Table 4). Most individuals invited agreed to participate. Due to the open nature of some community settings, it was not practically feasible to record the number of individuals who declined participation. Individuals who declined participation either indicated a lack of time or did not provide a reason. Five carers opportunistically participated in repeat interviews, which typically occurred when they had additional insights to share during interviews involving other members of their family or community.

**TABLE 4:** Participant numbers.

Site	Region, state	Participant numbers (carers)
1	Kimberley, Western Australia	42
2	Kimberley, Western Australia	
3	Kimberley, Western Australia	
4	Kimberley, Western Australia	57
5	Perth, Western Australia	16
6	Victoria Daly, Northern Territory	8
7	East Arnhem, Northern Territory	15
8	Darling Downs, Queensland	29
	Total number of carers	167

### 3.1 Findings

Key barriers and facilitators were organised into themes: (i) knowledge; (ii) beliefs and attitudes; (iii) perceived skills of clinicians; and (iv) clinic processes and environment. Figure 2 depicts a thematic map illustrating the classification of themes, subthemes and codes. The codes depicted were identified in all or most communities. Important codes unique to one or two communities are also discussed below.

**FIGURE 2:**
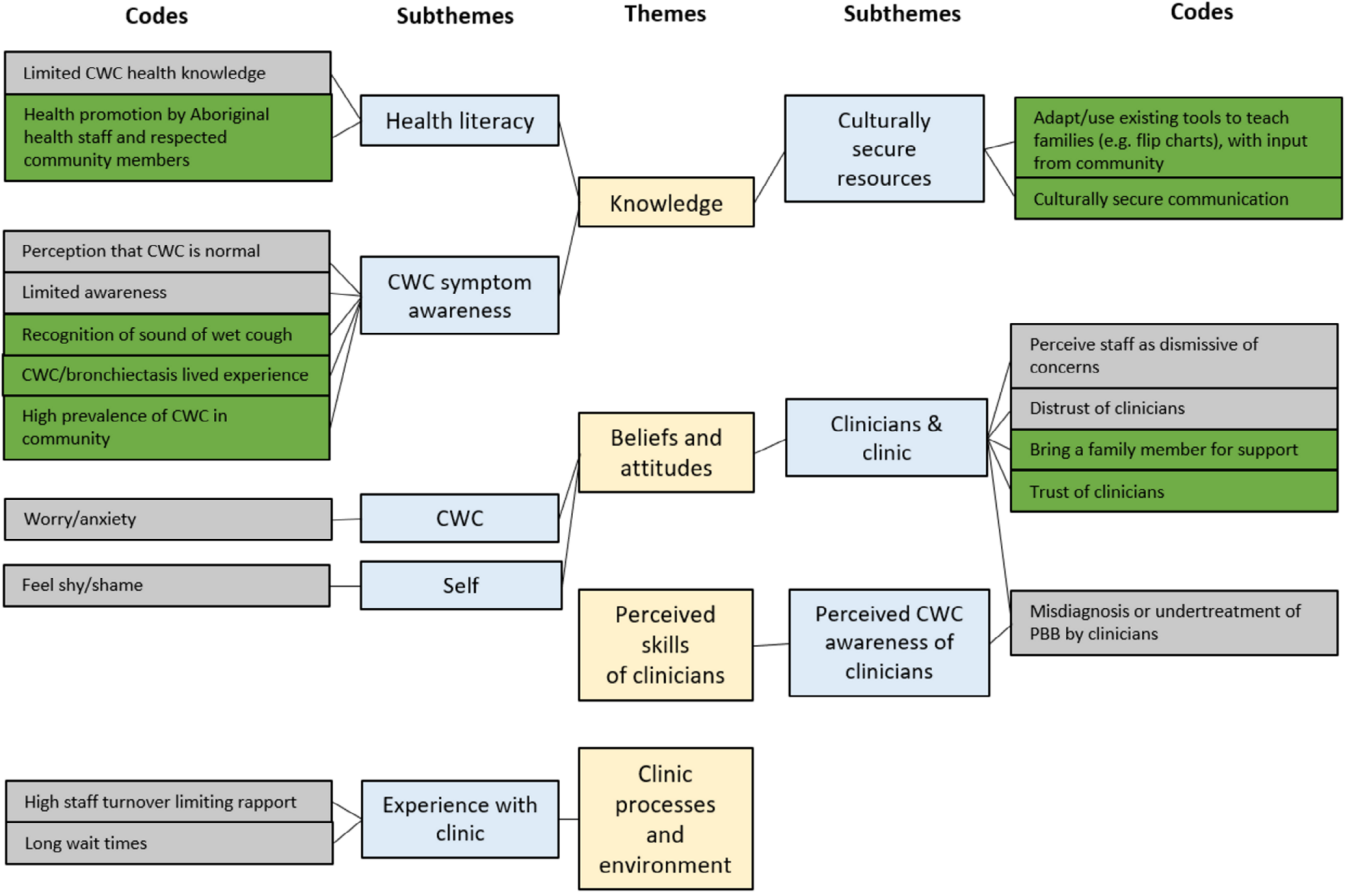
Thematic map of themes, subthemes and codes identified in most or all communities. CWC, chronic wet cough. Grey = barriers; green = facilitators.

### 3.2 Barriers to Timely Detection and Optimal Management of CWC

#### 3.2.1 Knowledge

##### 3.2.1.1 Health Literacy

Across all communities, limited knowledge about CWC was reported, with most carers stating they had never received information about it. A few carers from Western Australian communities reported familiarity with CWC because they had been exposed to previous CWC research in communities 700 km from the current study sites.

Right, so that makes so much sense. I never knew this. I was worried about the cough, but I haven't been able to put my finger on why I should be worried. Now, it makes sense. It's been difficult to get answers because she doesn't have a fever, and she doesn't struggle to breathe.(Mother, Community 5)

##### 3.2.1.2 CWC Symptom Awareness

Carers in all communities described the presence of CWC in children as normal. In some cases, this incorporated a reluctant acceptance of their child's usual health status in the context of the high prevalence of CWC in the community.

She's [daughter] like just learnt to just deal with it and continue on. Like, that's just her normal.(Mother, Community 8)

#### 3.2.2 Belief and Attitudes

##### 3.2.2.1 About Clinicians and Clinic

Carers expressed various feelings towards clinicians, ranging from appreciation to believing that concerns were overlooked. Some carers described mistrust of health staff, particularly when new clinicians had yet to establish strong connections or familiarity with local people.

You just feel like you're wasting time going to the doctors. They keep telling me it's fine. You just feel like they don't listen, kind of thing. It's so tiring … I got really frustrated and didn't take her to doctors anymore because they [the doctors] just keep saying virus after virus after virus.(Mother, Community 5)

##### 3.2.2.2 About CWC and Self

Carers reported feeling worried or anxious about their child's CWC but expressed reluctance and lacked confidence in advocating for their child at the clinic. Some carers shared that clinicians' questions about their child's CWC often made them feel uncomfortable or as though they were being judged.

Doctor was like “what, how long it's been?” like I thought I was a bad mother or something … I was like, “ah, well, what, have I done something wrong for bringing him in?” but then sometimes I would come in, but it would be just brushed off. (Mother, Community 8)

#### 3.2.3 Perceived Skills of Clinicians

##### 3.2.3.1 Perceived Knowledge of Clinicians

Some carers perceived clinicians had limited awareness about CWC.

When I took her to the doctor when she had the fever and breathing struggles, the doctors were great. But nobody seems to worry about the cough on its own. (Mother, Community 5)

#### 3.2.4 Clinic Processes and Environment

##### 3.2.4.1 Experience With the Clinic

Carers in most communities reported long wait times. Except in Queensland, high staff turnover was cited as a barrier, hindering rapport and trust between clinic staff and community members.

The doctors that have been here a long time are good too. But if there are different doctors, it gets a bit confusing because they tell you different things. But if the doctor knows our history, then it is easier. (Mother, Community 1)

In some very remote communities, practical issues, such as limited transportation, were identified as barriers preventing families from attending clinics.

### 3.3 Facilitators to Timely Detection and Optimal Management of CWC

#### 3.3.1 Knowledge

##### 3.3.1.1 Health Literacy and Symptom Awareness

The overwhelming message from carers in all communities was the need to improve health literacy through health promotion on CWC.

This is something that is important to our people. They need to hear the story. (Grandmother, Community 1)

Strong emphasis was placed on empowering local Aboriginal health staff for central roles in health promotion teams. Advice to improve health promotion was varied between communities. Smaller communities wanted door‐to‐door health promotion and community gatherings (e.g., barbeques), whereas larger communities wanted organised events accessible to families.

Health promotion team needs a local person to break community and cultural barriers. And a person in the team who knows all about CWC, an expert with full understanding to field questions … A local AHW [Aboriginal Health Worker] could be trained up as the expert. It's about building Indigenous people's capacity. Make them feel like they own it because it's their community. You get to build your own people up. (Mother, Community 4)

Radio, television and social media use varied across communities, influencing the suggested health promotion resources. In smaller communities, information was disseminated primarily through radio or word‐of‐mouth, whereas in larger communities with widespread cellular networks, social media was widely used.

Variations in the practical aspects of health promotion across different communities are summarised in Table 5.

**TABLE 5:** Health promotion across different communities.

Type of health promotion	Communities 1–4 (small, remote, or very remote communities)	Community 5 (large metropolitan city)	Communities 6–7 (small, remote, or very remote communities)	Community 8 (regional city)
Face‐to‐face health promotion by First Nations people	++	++	++	++
Door‐to‐door	++	—	+	—
Opportunistic yarning in community	++	—	++	—
Organised yarning circles at community groups (e.g., playgroups)	++	++	++	++
Radio health messages	++	+	+	++
Television health messages	+	+	—	+
Social media	+	++	+	++

Abbreviations: ++ = consistently identified as a facilitator, + = majority of participants identified this as a facilitator, — = not a facilitator.

##### 3.3.1.2 Culturally Secure Resources

Carers provided numerous suggestions for health promotion resources, emphasising need for culturally safe and visually engaging materials. Suggestions included printed posters, information flipcharts, video health messages, talking posters and banners. Carers highlighted the importance of incorporating the sound of a wet cough, suggesting that sound is a powerful educational tool for helping carers more effectively identify wet cough.

Aboriginal health TV in the [clinic] waiting rooms, put the video on that. Also, posters, something eye‐catching. It'd be good to have Aboriginal cartoons or artwork. (Mother, Community 4)

The suggested resource design elements varied across communities but emphasised enhancing relatability of visual components. Suggestions included incorporating recognisable local landmarks or using colour schemes that reflected the local natural environment. Some carers preferred to include local community members in resources, while others favoured featuring nationally known and respected Aboriginal figures.

Another facilitator identified in smaller communities was the desire for resources to include translations into the local language alongside English, recognising the linguistic diversity within these areas.

#### 3.3.2 Belief and Attitudes

##### 3.3.2.1 About Clinicians and Clinic

Carers explained that long‐term health staff fostered trust and strengthened the relationship between carers and healthcare providers. In addition, carers noted the importance of a high proportion of Aboriginal clinic staff for encouraging them to seek healthcare for their child. Some carers also identified that they would bring an older family member to help communicate their concerns when feeling too shy to speak to health staff.

Some doctors just make you feel like you're part of the family and really care about you. (Mother, Community 8)

## 4 Discussion

In this study exploring the barriers and facilitators to detecting and managing CWC from the perspectives of carers of First Nations children, interviews of 167 people across eight communities identified several key findings. Parents and carers from diverse Aboriginal Australian communities spanning multiple geographical regions and cultural identities highlighted two key barriers to the timely detection and management of CWC in children: inadequate health promotion and education and the widely held but incorrect belief that CWC in children is normal. High turnover among health staff also emerged as a significant issue, often leading to mistrust of newly arrived health staff. The overarching facilitator identified in all communities was knowledge dissemination through health promotion led by Aboriginal health staff using culturally secure resources tailored to each community. In addition, community members valued long‐term health staff with whom they developed trust and rapport.

The lack of health promotion as a barrier to appropriate health‐seeking for CWC along with the misconception that CWC is normal due to its high prevalence align with findings from previously published literature [16]. The challenges posed by limited health promotion and perception that CWC is normal due to high prevalence have been observed in other common primary care conditions in First Nations children, such as otitis media [24] and skin infections [25, 26], suggesting that a broader systemic issue may exist in managing medical conditions. These findings highlight the limitations of fragmented, condition‐specific health promotion strategies and underscore the need for coordinated, culturally secure and community‐led approaches that address shared underlying barriers to care.

Carers across all communities raised the need for culturally secure health promotion as a critical enabler for appropriate health‐seeking and effective management of CWC, with Aboriginal health staff central to the health promotion team. This aligns with existing literature on the importance of targeted health education and awareness campaigns to counteract the perception that CWC is normal and promote timely healthcare engagement [16]. Although preferences regarding the specific design of health promotion resources varied across community sites, carers consistently emphasised the need to connect and relate with the resources, underscoring the critical role of co‐creation in developing culturally relevant and impactful health promotion strategies [27]. Similar strategies have been reported as essential for addressing other prevalent conditions such as otitis media [24] and oral health [28] in First Nations communities.

High staff turnover emerged as a pervasive barrier due to its detrimental impact on building rapport and trust between healthcare workers and community members. The challenge of retaining health workers in rural and remote Australia is well‐documented [29], with reported average annual turnover rates of 151% in regional and remote Aboriginal Community Controlled Health Service clinics in Western Australia and the Northern Territory [30]. However, the impact of high staff turnover on community trust and the subsequent reduction in health‐seeking is less well described. Carers highlighted that long‐term staff were instrumental in fostering trust and rapport, emphasising the need for sustained workforce retention initiatives. Despite the introduction of numerous government incentives to improve staff retention in remote communities [31], the effectiveness of these incentives remains to be seen. Community‐led initiatives that strengthen connections between staff and the community may offer complementary strategies, fostering trust, morale and a sense of belonging, which could in turn support workforce stability.

The main findings in our study align with multiple smaller studies conducted in First Nation communities across different countries and various health conditions [16, 25, 26, 32, 33, 34, 35]. Our study is important as the first comprehensive assessment of the barriers and facilitators across multiple diverse settings while undertaking research that impacts health outcomes, grounded in a framework that prioritises Aboriginal voices and close collaboration with the people affected [6]. This broad scope strengthens the relevance and applicability of our results to inform culturally secure strategies and support the implementation of feasible and sustainable healthcare initiatives that are community‐driven and align with the needs of Aboriginal families.

### 4.1 Limitations

A potential limitation is that recruitment through health clinics may have resulted in over‐representation of families who engage with community and health services and exclusion of people hesitant to engage with health services. However, we recruited via referrals from other families, conducted home visits and visited community settings (e.g., playgroups), helping ensure a broader participant group. We tried to reach a diverse range of families by engaging geographically diverse regions and cultural groups and observed similar barriers across sites. Our approach allowed us to capture a broad range of perspectives and ensured that identified challenges were not isolated to specific communities or service‐engaged families. Another potential limitation is that Aboriginal families may have been reluctant to discuss specific barriers with non‐Aboriginal interviewers, such as socio‐cultural influences on detection and management of CWC. Conducting interviews solely in English may have limited the ability of some participants to fully express their perspectives, particularly given the diversity of local languages across the eight communities. However, English was commonly used in the participating communities, and over 90% of interviews included a local Aboriginal research team member, supporting culturally secure communication and helping facilitate understanding across different language contexts.

### 4.2 Conclusion

Barriers and facilitators to timely detection and optimal management of CWC were broadly consistent across cultural and geographical contexts in Australia. Aboriginal families wanted Aboriginal‐led face‐to‐face health promotion with culturally secure visual resources. Families also emphasised the importance of regular clinic staffing in fostering trust and rapport between clinic staff and the community.

Given the high rates of CWC and high burden of chronic lung disease among First Nations people [2, 4], urgent action is required to develop and implement these community‐identified strategies. A co‐design approach involving First Nations communities is crucial for developing locally tailored solutions and ensuring the cultural relevance and effectiveness of health initiatives. Further, efforts to reduce staff turnover in primary care clinics cannot be underestimated.

## Author Contributions

Conceptualisation: A.S., R.W., A.B.C. Data curation: G.T.Y.L., A.F., A.S. Formal analysis: G.T.Y.L., M.K., M.T., P.L., R.A., E.R.B., N.J., G.B.M., A.S. Funding acquisition: A.S., A.B.C., R.A., G.B.M., P.M., R.N., M.T., R.W., A.B.C. Investigation: G.T.Y.L., R.A., L.B., P.L., E.R.B., M.K., G.B.M., S.S., M.T., J.E., R.K., C.T., J.W., Aa.S., An.S. Methodology: An.S., A.B.C., R.A., G.B.M., P.M., R.N., M.T., R.W., A.B.C. Project administration: G.T.Y.L., A.F., An.S. Supervision: An.S., R.A., G.B.M., A.B.C., R.W. Writing (original draft): G.T.Y.L. Writing (review and editing): All authors.

## Funding

A National Health and Medical Research Council partnership grant (1170735) funded the research expenditure costs and salaries of program managers and local researchers. A.S. received salary support from Medical Research Future Fund Investigator Grant (1193796). A.B.C. received salary support from National Health and Research Council Investigator Grant (2025379). The funding sources had no role in planning, writing or publication of the work, or for a research paper, any role in study design, data collection, analysis or interpretation, reporting or publication.

## Conflicts of Interest

The authors declare no conflicts of interest.

## Supporting information


**Section S1:** CONSolIDated critERia for strengthening the reporting of health research involving Indigenous Peoples (CONSIDER).
**Box S1:** Interview guide.
**Table S1:** COREQ (COnsolidated criteria for REporting Qualitative research) Checklist.

## Data Availability

The participants of this study did not give written consent for their data to be shared publicly, so due to the sensitive nature of the research, supporting data are not available.
