# Supplementary material for: Barriers and Facilitators to Timely Detection and Optimal Management of Chronic Wet Cough in Aboriginal Children Across Australia From the Perspectives of Carers of Aboriginal Children: A Qualitative Study
Source: Med J Aust. 2026 Oct 1;224(10):e70313. doi: 10.5694/mja2.70313 (PMC13630464; doi:10.5694/mja2.70313)
Supplement: Supplementary file 1 [file MJA2-224-0-s001.pdf]

MJA
The Medical Journal of Australia

Supporting Information

Supplementary material

This appendix was part of the submitted manuscript and has been peer reviewed.
It is posted as supplied by the authors.

Appendix to: G. T. Y. Lau, P. Laird, R. Aitken, et al. Barriers and Facilitators to Timely Detection and Optimal Management of Chronic Wet Cough in Aboriginal Children Across Australia From the Perspectives of Carers of Aboriginal Children: A Qualitative Study. *Medical Journal of Australia* 2026; doi: 10.5694/mja2.70313.

SECTION S1. CONSolIDated critERia for strengthening the reporting of health research involving Indigenous Peoples (CONSIDER)

Governance

1. Describe partnership agreements between the research institution and Indigenous-governing organization for the research, (e.g., Informal agreements through to MOU (Memorandum of Understanding) or MOA (Memorandum of Agreement)).
   - Formal Multi-Institutional Agreement (MIA)
   - Letter of Support

2. Describe accountability and review mechanisms within the partnership agreement that addresses harm minimization.
   - Project is conducted and governed in accordance with NHMRC Approved Standards and Guidelines.
   - The project was approved by the Western Australian Health Child and Adolescent Health Service Ethics Committee, Western Australian Aboriginal Health Ethics Committee, Human Research Ethics Committee of the Northern Territory Department of Health and Menzies School of Health Research, Miwatj Research and Ethics Advisory Group, and reciprocal ethics was given by the University of Queensland Human Research Ethics Committee.
   - All confidential information disclosed by one party to the other will be kept confidential and shall not be disclosed to any third party without the prior written consent of the disclosing party.

3. Specify how the research partnership agreement includes protection of Indigenous intellectual property and knowledge arising from the research, including financial and intellectual benefits generated (e.g., development of traditional medicines for commercial purposes or supporting the Indigenous community to develop commercialization proposals generated from the research).
   - Co-designed project with Indigenous organisations and leaders.
   - Knowledge generated will be leading to adaption and generation of strategies to overcome the barriers identified, thereby facilitating timely health care for Indigenous children.
   - Indigenous community will directly benefit through development of local resources, provision of culturally secure health information for families, and training of local clinicians.
   - Each party grants to each other party a royalty-free, non-exclusive, non-transferrable licence to use the project intellectual property for non-commercial research, education and training purposes, and publication purposes
   - Co-authorship with Indigenous research partners.
   - Resources will be developed together with the Indigenous community and be freely available to use thereafter.

Prioritization

4. Explain how the research aims emerged from priorities identified by either Indigenous stakeholders, governing bodies, funders, non-government organization(s), stakeholders, consumers, and empirical evidence

- Indigenous community members in the East Kimberley and Darling Downs communities saw the benefits from our previous research on chronic wet cough in another town and asked if the project could be extended to their communities.
- The study prioritises mutual benefit by fostering skills development, knowledge-sharing and opportunities for Indigenous co-designed research.
- Chronic wet cough remains a significant health issue in the Australian Indigenous children, as it is often an indicator of protracted bacterial bronchitis, which if left untreated, can lead to life-limiting disease (bronchiectasis).

Relationships (Indigenous stakeholders/participants and Research team)
5. Specify measures that adhere and honor Indigenous ethical guidelines, processes, and approvals for all relevant Indigenous stakeholders, recognizing that multiple Indigenous partners may be involved, e.g., Indigenous ethics committee approval, regional/national ethics approval processes.

- The project has had ethical approval from all relevant Indigenous committees, as described in item 2.

6. Report how Indigenous stakeholders were involved in the research processes (i.e., research design, funding, implementation, analysis, dissemination/recruitment).

- Grant proposal that funded this study included Indigenous CIs.
- Research plan was co-designed with Indigenous stakeholders and leaders.
- Indigenous research partners were present and facilitated the recruitment and interview process (data collection).
- Indigenous stakeholders contributed to interpretation of results and reviewed the findings, data dissemination and manuscript.

7. Describe the expertise of the research team in Indigenous health and research.

- Many of the research team are active contributors to AusBREATHE ("Australian Bronchiectasis Centre of Research Excellence especially for Aboriginal and Torres Strait Islander Children"),[1] an NHMRC Centre of Research Excellence in Bronchiectasis, including leadership group members and chief investigators. The CRE leads nationally coordinated research, capacity building, advocacy and knowledge translation activities aimed at reducing the burden of bronchiectasis and improving respiratory health outcomes for Aboriginal and Torres Strait Islander children and other children at high risk of developing bronchiectasis.
- The research team brings extensive expertise and leadership in Indigenous child health research, with a strong track record in research demonstrated through major collaborative projects and numerous peer-reviewed publications, with a small selection referenced here.[2-18] The team has longstanding experience working in partnership with Aboriginal and Torres Strait Islander communities, health services and clinicians to deliver culturally responsive, community-informed research with a strong focus on translation into practice and policy.
- Importantly, the research team includes Indigenous researchers and collaborators who contribute essential cultural knowledge, lived experience and community perspectives to the design and conduct of research, and the qualitative analysis of findings. Their partnership and guidance help ensure that the research is culturally secure, community-informed and aligned with the priorities and strengths of Aboriginal and Torres Strait Islander peoples. The team is committed to respectful partnership

approaches that support Indigenous leadership, strengthen research capacity and promote meaningful and sustainable benefits for communities.

Methodologies

8. Describe the methodological approach of the research including a rationale of methods used and implication for Indigenous stakeholders, e.g., privacy and confidentiality (individual and collective)

- A co-designed participatory action research[19] project to ensure Indigenous voice and values are central to the methodology.
- Adherence to Indigenous data sovereignty principles, ensuring data collection, storage and sharing align with community expectations and governance protocols.

9. Describe how the research methodology incorporated consideration of the physical, social, economic and cultural environment of the participants and prospective participants. (e.g., impacts of colonization, racism, and social justice). As well as Indigenous worldviews.

- The project was shaped by Indigenous ways of knowing, being, and doing. It prioritises storytelling, yarning circles[20] and culturally appropriate research methods to respect relational knowledge-sharing.
- The study acknowledges the ongoing effects of systemic racism and intergenerational trauma on Indigenous communities and actively seeks to challenge power imbalances through research that was co-designed and accomplished with Indigenous partners from study design to reporting.
- The research findings are designed to be accessible and useful for Indigenous communities, supporting advocacy efforts, policy development, and community-driven solutions.

Participation

10. Specify how individual and collective consent was sought to conduct future analysis on collected samples and data (e.g., additional secondary analyses; third-parties accessing samples (genetic, tissue, blood) for further analyses).

- Qualitative data
    - Informed consent was obtained from individuals at the time of the interviews/focus groups and surveys.
    - Data are de-identified while respecting collective ownership of knowledge.
- Quantitative data
    - A waiver of consent was granted by all relevant ethics committees for medical record audits in the two-6-month periods pre-and post-intervention.

11. Described how the resource demands (current and future) placed on Indigenous participants and communities involved in the research were identified and agreed upon including any resourcing for participation, knowledge, and expertise

- Early engagement and consultation – extensive consultation with Indigenous stakeholders and community representatives were undertaken to identify potential resource demands.
- The study was designed in collaboration with the local and regional Aboriginal Medical Services to ensure that participation expectations are reasonable, culturally appropriate, and aligned with community priorities.

- Indigenous Elders, knowledge holders, and community members engaged as co-researchers or advisors receive formal recognition and fair compensation for their intellectual contributions.
- Findings and outcomes are shared back with communities in accessible formats, ensuring that the research provides lasting benefits and informs community-led solutions.

12. Specify how biological tissue and other samples including data were stored, explaining the processes of removal from traditional lands, if done, and of disposal.

- N/A

Capacity

13. Explain how the research supported the development and maintenance of Indigenous research capacity (e.g., specific funding of Indigenous researchers).

- Indigenous Elders, knowledge holders, and community members engaged as co-researchers or advisors received formal recognition through co-authorship and compensation for their intellectual contributions.
- The research paper support Indigenous capacity by arguing for expanding the scope of Indigenous practitioners.

14. Discuss how the research team undertook professional development opportunities to develop the capacity to partner with Indigenous stakeholders?

- The research team have participated in cultural safety and awareness training, facilitated by Indigenous leaders.
- The research team also priorities on-Country learning experiences with Indigenous Elders and stakeholders – these immersive experiences have been critical to foster trust, build relationships and ensure the team's approach is grounded in cultural respect and reciprocity.
- Indigenous researchers and community leaders were engaged as mentors and co-designers throughout the project, providing guidance on culturally appropriate methodologies, protocols and relationship-building strategies.
- Professional development is viewed as an ongoing process, and feedback from Indigenous participants and community partners were actively sought and incorporated.

Analysis and interpretation

15. Specify how the research analysis and reporting supported critical inquiry and a strength-based approach that was inclusive of Indigenous values.

- A strength-based approach was embedded throughout the analysis and reporting by highlighting community existing strengths and positive practices rather than focusing solely on deficits. Indigenous values were upheld by prioritising respect, reciprocity, and community voice, and by ensuring that findings were communicated in a culturally appropriate and accessible manner.
- The data, and the implementation process that followed, were presented to key community members at each site, allowing for review, validation, and further feedback. This iterative process supported critical inquiry by enabling community perspectives to shape the interpretation of findings and ensured that conclusions were grounded in local knowledge and context.

Dissemination
16. Describe the dissemination of the research findings to relevant Indigenous governing bodies and peoples.

- The research analysis and reporting in this study effectively supported critical inquiry and a strength-based approach that was inclusive of Indigenous values in several ways:
- **Collaborative and Co-Designed Approach**: The study employed an Aboriginal co-designed, participatory action research[19] methodology. This approach ensured that Aboriginal communities, particularly through the involvement of Indigenous researchers and stakeholders, were integral to the research process. It reflects a strength-based approach by valuing Indigenous knowledge systems and experiences, and empowering communities to actively contribute to the design and direction of the research.
- **Focus on Strengths and Resources**: Rather than focusing solely on barriers, the study highlighted facilitators, including the role of Aboriginal Health Practitioners (AHPs) and the importance of building capacity among health care providers (HCPs) through training. A strength-based approach is seen in the identification of practical solutions to enhance clinical management. This acknowledges the existing capabilities within the Indigenous health workforce and promotes leveraging local expertise as a way to improve outcomes.
- **Inclusion of Indigenous Perspectives**: Incorporating the perspectives of Aboriginal HCPs and Indigenous families in the research was inclusive of Indigenous values. The research did not just observe the issue from a Western medical model but considered how Indigenous practitioners and community members might have culturally appropriate insights into the timely detection and management of chronic wet cough. This approach ensures the results are relevant and culturally sensitive.
- **Application of Indigenous Frameworks**: The study employed a participatory action research[19] design that prioritizes Indigenous knowledge and participation. This aligns with Indigenous values of community involvement, self-determination, and collective action. Such frameworks ensure that the research does not merely extract data from Indigenous communities but works in partnership to promote community empowerment.
- **Critical Inquiry into Barriers and Solutions**: The thematic analysis was based on the identification of both barriers and facilitators, giving a balanced view that acknowledges challenges while also proposing solutions. This inquiry process encourages an exploration of complex issues with respect to context.
- **Policy and System-Level Implications**: The study proposed clinic system changes to improve the timely management of CWC, such as visible guidelines, empowering nurses and AHPs to manage CWC, and electronic decision record supports. These recommendations align with a critical, systems-thinking approach to address root causes of inequity in health care access and quality. The research doesn’t just point to individual behaviour change but calls for structural and policy adjustments which demonstrates respect for Indigenous expertise and values.
- In summary, the study is a strong example of integrating critical inquiry and a strength-based approach that is inclusive of Indigenous values. It recognizes the importance of community engagement, respects Indigenous knowledge, and advocates for systemic solutions that empower Indigenous health workers and communities.

17. Discuss the process for knowledge translation and implementation to support Indigenous advancement (e.g., research capacity, policy, investment).

- This project addresses a large gap in an area of health where potential gains are large and life-long through improved lung health trajectories and reducing the burden of chronic respiratory disease.
- The project will support Indigenous advancement through the translation of knowledge and skills, while building the capacity of local Aboriginal community members, health workers, and clinicians to lead and sustain improvements in child lung health.
- Findings from this work will inform future policy, creation of culturally secure resources, service delivery and design, and improvement in culturally secure models of care for Indigenous children and communities.

References

1. Menzies School of Health Research. The CRE in Lung Health Especially for First Nations Children 2020; cited 2026 22 May]. https://crelungs.org.au/
2. Laird P, Walker R, Lane M, Totterdell J, Chang AB, Schultz A. Recognition and Management of Protracted Bacterial Bronchitis in Australian Aboriginal Children: A Knowledge Translation Approach. CHEST. 2021;159(1):249-58.
3. Lau GTY, Walker R, Laird P, Lewis P, Kuthubutheen J, Schultz A. Identifying barriers and facilitators for the effective diagnosis and provision of primary health care for otitis media from the perspective of carers of Aboriginal children. J Paediatr Child Health. 2024;60:505-10.
4. Lau GTY, Walker R, Laird P, Lewis P, Kuthubutheen J, Schultz A. The determinants to effective diagnosis and management of otitis media in Aboriginal children in primary care from the perspective of health care practitioners. Aust J Rural Health. 2025;34:e70144.
5. Chang; AB, Kovesi; T, Redding; GJ, et al. Improving the respiratory health of Indigenous peoples globally - the need, why and how. . Lancet Respir Med. 2024;12(7):556-74.
6. Schultz A, Chang AB, Mason DM, et al. Guiding practice principles for clinicians who work with Indigenous peoples. Frontiers. 2025;13.
7. Laird P, Chang AB, Jacky J, Lane M, Schultz A, Walker R. Conducting decolonizing research and practice with Australian First Nations to close the health gap. Health Res Policy Sys. 2021;19(127).
8. Laird PJ, Chang AB, Walker R, et al. Evaluation of the implementation and clinicial effects of an intervention to improve medical follow-up and health outcomes for Aboriginal children hospitalised with chest infections. Lancet Reg Health West Pac. 2023;34.
9. Toombs MR, Curtis C, Brolan CE. Supporting Indigenous health equity strategic planning: a Queensland perspective. Med J Aust. 2023;218(1):5-8.
10. Bowden ER, Toombs MR, Chang AB, McCallum GB, Williams RL. 'Walking the journey' with pregnant and birthing women from remote Australian First Nations communities: A qualitative study in the Top End of the Northern Territory. Midwifery. 2025;141.
11. D'Sylva P, Walker R, Lane M, Chang AB, Schultz A. Chronic wet cough in Aboriginal children: It's not just a cough. J Paediatr Child Health. 2018;55:833-43.
12. Laird P, Totterdell J, Walker R, Chang AB, Schultz A. Prevalence of chronic wet cough and PBB in Aboriginal children. ERJ Open Res. 2019;5:00248-2019.
13. Laird P, Walker R, Lane M, Chang AB, Schultz A. We won't find what we don't look for: Identifying barriers and enablers of chronic wet cough in Aboriginal children. Respirology. 2020;25:383-92.

14. Laird; PJ, Walker; R, McCallum; G, et al. Change in health outcomes for First Nations children with chronic wet cough: rationale and study protocol for a multi-centre implementation science study. BMC Pulm Med. 2022;22:492.
15. Laird P, Walker R, Lane M, Chang AB, Schultz A. Educational Resource, Chronic Lung Sickness. University of Western Australia: Telethon Kids Institute; 2018.
16. Bowden ER, Toombs MR, Chang AB, McCallum GB, Williams RL. Listening to First Nations women's voices, hearing requests for continuity of carer, trusted knowledge and family involvement: A qualitative study in urban Darwin. Women Birth. 2023;36(5).
17. McCallum GB, Chang AB. 'Good enough' is 'not enough' when managing Indigenous adults with bronchiectasis in Australia and New Zealand. Respirology. 2018;23(8):725-6.
18. Chang AB, Kovesi T, Redding GJ, et al. Chronic respiratory disease in Indigenous peoples: a frameowrk to address inequity and strengthen respiratory health and health care globally. Lancet Respir Med. 2024;12(7):556-74.
19. Dudgeon P, Bray A, Darlaston-Jones D, Walker R. Aboriginal Participatory Action Research: An Indigenous Research Methodology Strengthening Decolonisation and Social and Emotional Wellbeing, Discussion paper. Melbourne: Lowitja Publishing; 2020.
20. Jennings W, Spurling GK, Askew DA. Yarning about health checks: barriers and enablers in an urban Aboriginal medical service. Aust J Prim Health. 2014;20(2):151-7.

BOX S1. Interview guide.

Barriers and Enablers to detection and management of chronic wet cough

1. Has your child ever had cold sick or hot sick with a cough?
    a. If yes, can you tell me a bit about what that was like?
2. Have you ever noticed a cough that just doesn't go away?
3. Has your bubs/child ever had a cough that sounds like there is spit/phlegm that goes on for a long time, but they don't have a fever, or trouble breathing?
    a. If yes, did you go the doctor or the clinic?
    b. If no, (Q4)
4. When would you see a doctor for your child's cough?
    a. If yes, when else would you go to the doctor for your child's cough?
5. Do you know about (or has someone (a nurse or doctor)) talked to you about wet and dry cough
6. Have you ever heard about wet cough and how lung damage might happen if the wet cough goes on for a long time?
7. Have you ever taken your children to the doctor or hospital with wet cough?
    a. If yes, (Q8)
8. How confident are you when managing ongoing wet cough?
9. Have you ever heard or been told how long a cough could go on for before you should see a doctor?
10. Would you feel confident to take your child to the doctor if they had a wet cough for a long time without a fever or other symptoms?
11. Has any clinic staff ever talked to you about lung health before?
12. Have you ever had any problems getting help at the clinic for your child if they had ongoing wet cough?
    a. If yes, (Q13)
13. What would help you or make it easier for you to see a doctor for your child's cough?

Barriers and Enablers to Health information campaign:

1. What do you think of the following information resources that we have? Would they be acceptable in your community? What would you change to make it work better in your community?
2. How can we best spread the word about chronic wet cough in your community?
    a. Specifically ask about social media, radio, television, other.
3. Ask about whether resources in English are appropriate, or should they be translated? If so, into which language(s)?

TABLE S1. COREQ (COnsolidated criteria for REporting Qualitative research) Checklist

A checklist of items that should be included in reports of qualitative research. You must report the page number in your manuscript where you consider each of the items listed in this checklist. If you have not included this information, either revise your manuscript accordingly before submitting or note N/A.

Topic	Item No.	Guide Questions/Description	Reported on Page No.
**Domain 1: Research team and reflexivity**			
Personal characteristics			
Interviewer/facilitator	1	Which author/s conducted the interview or focus group?	Table 3
Credentials	2	What were the researcher's credentials? E.g. PhD, MD	Table 3
Occupation	3	What was their occupation at the time of the study?	Table 3
Gender	4	Was the researcher male or female?	Table 3
Experience and training	5	What experience or training did the researcher have?	Table 3
Relationship with participants			
Relationship established	6	Was a relationship established prior to study commencement?	4-5
Participant knowledge of the interviewer	7	What did the participants know about the researcher? e.g. personal goals, reasons for doing the research	4-5
Interviewer characteristics	8	What characteristics were reported about the inter viewer/facilitator? e.g. Bias, assumptions, reasons and interests in the research topic	4-5
**Domain 2: Study design**			
Theoretical framework			
Methodological orientation and Theory	9	What methodological orientation was stated to underpin the study? e.g. grounded theory, discourse analysis, ethnography, phenomenology, content analysis	4
Participant selection			
Sampling	10	How were participants selected? e.g. purposive, convenience, consecutive, snowball	5
Method of approach	11	How were participants approached? e.g. face-to-face, telephone, mail, email	5
Sample size	12	How many participants were in the study?	7
Non-participation	13	How many people refused to participate or dropped out? Reasons?	7
Setting			
Setting of data collection	14	Where was the data collected? e.g. home, clinic, workplace	5-6
Presence of non-participants	15	Was anyone else present besides the participants and researchers?	5-6
Description of sample	16	What are the important characteristics of the sample? e.g. demographic data, date	7, Table 4
Data collection			
Interview guide	17	Were questions, prompts, guides provided by the authors? Was it pilot tested?	5, Appendix
Repeat interviews	18	Were repeat inter views carried out? If yes, how many?	7
Audio/visual recording	19	Did the research use audio or visual recording to collect the data?	5-6
Field notes	20	Were field notes made during and/or after the inter view or focus group?	5-6
Duration	21	What was the duration of the inter views or focus group?	5-6
Data saturation	22	Was data saturation discussed?	5
Transcripts returned	23	Were transcripts returned to participants for comment and/or	5-6

Topic	Item No.	Guide Questions/Description	Reported on Page No.
		correction?	
**Domain 3: analysis and findings**			
Data analysis			
Number of data coders	24	How many data coders coded the data?	6
Description of the coding tree	25	Did authors provide a description of the coding tree?	7, Figure 2
Derivation of themes	26	Were themes identified in advance or derived from the data?	6
Software	27	What software, if applicable, was used to manage the data?	6
Participant checking	28	Did participants provide feedback on the findings?	6
Reporting			
Quotations presented	29	Were participant quotations presented to illustrate the themes/findings? Was each quotation identified? e.g. participant number	7-11
Data and findings consistent	30	Was there consistency between the data presented and the findings?	7-13
Clarity of major themes	31	Were major themes clearly presented in the findings?	7-13
Clarity of minor themes	32	Is there a description of diverse cases or discussion of minor themes?	11-12

Developed from: Tong A, Sainsbury P, Craig J. Consolidated criteria for reporting qualitative research (COREQ): a 32-item checklist for interviews and focus groups. *International Journal for Quality in Health Care*. 2007. Volume 19, Number 6: pp. 349 – 357

Once you have completed this checklist, please save a copy and upload it as part of your submission. DO NOT include this checklist as part of the main manuscript document. It must be uploaded as a separate file.
